# Data-independent proteome profile of *Mycoplasma gallisepticum* under normal conditions and heat stress

**DOI:** 10.1016/j.dib.2017.11.093

**Published:** 2017-12-07

**Authors:** Ivan Butenko, Olga Pobeguts, Daria Matyushkina, Sergey Kovalchuk, Nickolay Anikanov, Gleb Fisunov, Vadim Govorun

**Affiliations:** Federal Research and Clinical Center of Physical-Chemical Medicine, Russian Federation

## Abstract

The data reported is a large-scale untargeted proteome profile for *Mycoplasma gallisepticum* – a model organism for studying both regulation in genome-reduced bacteria and intracellular infection (Mazin et al., 2014) [1,2]. While seminal whole-proteome studies were performed on *Mycoplasma genitalium* [3] and a few proteome datasets are available for *Mycoplasma pneumoniae*, no data-independent (DIA) proteome profiling has been published for bacteria of Mycoplasma genus. Since DIA-based proteome profiling allows to extract evidence on presence and quantity of any protein of interest in a post-acquisition manner and the data presented is describing a model which is suitable to study both proteome regulation in general and details of mycoplasma infection process [4], the proteome profiling data presented here is of value for deep annotation. The data was deposited to the PRIDE repository (PXD008198).

**Specifications Table**

**Table:** 

Subject area	Biology
More specific subject area	Systems biology of model minimal cell; proteomics;
Type of data	LC-MS/MS data
How data was acquired	Data-independent acquisition (SWATH analysis), data-dependent acquisition (IDA/DDA) using Sciex TripleTOF 5600+ QTOF mass-spectrometer.
Data format	Raw
Experimental factors	*Mycoplasma gallisepticum* was cultivated under standard laboratory conditions as described previously [5] and subjected to sublethal heat stress. Cells were sampled before the stress, immediately after it, and after additional cultivation. Samples were processed by common bottom-up proteomics protocol and subjected to LC-MS/MS analysis.
Experimental features	*Mycoplasma gallisepticum* was subjected to sublethal heat shock. Protein extraction and trypsin digestion in solution
	were performed as described previously [2].
Data source location	Research and Clinical Center of Physical-Chemical Medicine, Moscow, Russian Federation
Data accessibility	Data was deposited to the PRIDE repository:
	https://www.ebi.ac.uk/pride/archive/projects/PXD008198
Related research article	Butenko et al. [4].

**Value of the data**

• The data itself is first publically available DIA-based proteome map of a member of Mollicutes genus.
• This data set will be of value for the scientific community working in the area of host-pathogen interaction since it represents the protein response of bacteria to heat stress (one of host inflammation reaction to bacterial infection).
• The data might be useful for deep annotation of *Mycoplasma gallisepticum* proteome in terms of unconventional proteoforms or post-translational modifications.
• The data might be used in studies considering mechanisms of regulation in minimal cell.

## 1 Data

Protein expression was assessed in untargeted label-free bottom-up proteomic experiment, where data was acquired using data-independent approach (i.e. SWATH - Sequential Window acquisition of All Theoretical fragment ion spectra) on Sciex TripleTOF 5600+ Q-TOF mass-spectrometer. Dataset covers 54 samples (biological replicates of control and treated mycoplasma), each analyzed with DIA acquisition in triplicate. SWATH data is accompanied with 6 data-dependent runs and identification results that allow building spectral library and extracting protein abundance data with tools of choice.

## 2 Experimental design, materials, and methods

### 2.1 Cell cultures

*Mycoplasma gallisepticum* stain S6 was cultivated at 37 °C on a modified Edwards medium (20 g/L tryptose, 3 g/L tris, 5 g/L NaCl, 5 g/L KCl, 5% yeast dialysate, 10% horse serum, 1% glucose) at pH 7.4 in aerobic conditions for 12 hours before heat chock. The cells were exposed to sublethal heat shock, i.e. cells were cultivated at 46 °C for 30 min and then conditioned for 2 h at 37 °C and compared to initial culture sampled right before inducing heat shock. Cells were harvested by centrifugation at 8000 rcf and 4 °C for 10 min. Stress conditions were elaborated in previous work [1]. Sample description is provided in Table 1.

**Table 1:** Sample description.

**Sample**	**Case**
T615	Control culture
T616	Control culture
T617	Control culture
T618	Control culture
T619	Control culture
T620	Control culture
T725	Control culture
T726	Control culture
T731	Control culture
T732	Control culture
T771	Control culture
T772	Control culture
T793	Control culture
T805	Control culture
T806	Control culture
T811	Control culture
T814	Control culture
T815	Control culture
T627	Culture 120 min after heat shock
T628	Culture 120 min after heat shock
T629	Culture 120 min after heat shock
T630	Culture 120 min after heat shock
T631	Culture 120 min after heat shock
T632	Culture 120 min after heat shock
T735	Culture 120 min after heat shock
T736	Culture 120 min after heat shock
T777	Culture 120 min after heat shock
T778	Culture 120 min after heat shock
T797	Culture 120 min after heat shock
T809	Culture 120 min after heat shock
T810	Culture 120 min after heat shock
T813	Culture 120 min after heat shock
T818	Culture 120 min after heat shock
T819	Culture 120 min after heat shock
T621	Culture 30 min after heat shock
T622	Culture 30 min after heat shock
T623	Culture 30 min after heat shock
T624	Culture 30 min after heat shock
T625	Culture 30 min after heat shock
T626	Culture 30 min after heat shock
T727	Culture 30 min after heat shock
T728	Culture 30 min after heat shock
T729	Culture 30 min after heat shock
T730	Culture 30 min after heat shock
T733	Culture 30 min after heat shock
T734	Culture 30 min after heat shock
T774	Culture 30 min after heat shock
T775	Culture 30 min after heat shock
T795	Culture 30 min after heat shock
T807	Culture 30 min after heat shock
T808	Culture 30 min after heat shock
T812	Culture 30 min after heat shock
T816	Culture 30 min after heat shock
T817	Culture 30 min after heat shock

### 2.2 Trypsin digestion in solution

Сells were washed three times with PBS, pH 7.5. Cell pellet was treated with 3 μl of 10% RapiGest SF (Waters) and 1 μl nuclease mix for 30 min at 4 °C, then resuspended in 37 μl of 100 mM NH_4_HCO_3_, vortexed and heated at 100 °C for 5 min. After cooling to room temperature cell debris was removed by centrifugation at 15,000 g for 5 min. Protein cysteine bonds were reduced with 10 mM DTT in 5 mM NH_4_HCO_3_ for 30 min at 60 °C and alkylated with 30 mM iodoacetamide in the dark at RT for 30 min. The step with adding DTT was repeated. Clarified extract protein concentration was estimated using Bradford Protein Assay Kit (BioRad). Trypsin (Trypsin Gold, Mass Spectrometry Grade, Promega) was added in 1/50 w/w trypsin/protein ratio and incubated at 37 °C overnight. To stop trypsinolysis and degrade the acid-labile RapiGest surfactant, trifluoroacetic acid (TFA) was added to the final concentration of 0,5% v/v (the pH should be less than 2.0), incubated at 37 °C for 45 min and the samples were centrifugated at 15,000 g for 10 min to remove the surfactant. Hydrolyzate was desalted using a Discovery DSC-18 Tube (Supelco) according to the manufacturer protocol. Peptides were eluted with 700 μL 75% acetonitrile, 0.1% TFA, dried in a SpeedVac (Labconco) and resuspended in 3% acetonitrile, 0.1% TFA to the final concentration of 5 μg/μL.

### 2.3 Protein identification

To generate spectral library for further quantitative analysis 6 information-dependent acquisition runs were performed on Sciex TripleTOF 5600+ QTOF mass-spectrometer coupled to Eksigent NanoLC Ultra 2D+ nano-HPLC system configured in trap-elute mode through Sciex NanoSpray III nano-ESI ion source. The gradient was from 5% to 40% of acetonitrile in water with 1% methanol and 0.1% formic acid, gradient length was 2 h with flowrate 300 nL/min. Eksigent 3C18-CL-120 column (3 μm, 120 Å 75 μm × 150 mm) was used for separation and Eksigent Chrom XP C18 trap column (3 μm, 120 Å 350 μm × 0.5 mm) was used for sample loading. Each survey MS spectrum was accumulated for 250 ms, each fragmentation spectrum was accumulated for 100 ms, collision energy was set to ramp from 25 to 55 eV during fragment spectrum acquisition. No more than 50 most abundant ions with intensity above 200 counts per second were subjected to MS/MS in each cycle, after which they were ignored in subsequent cycles for 15 s.

Identification was performed with Sciex ProteinPilot 4.5 software against a database of all proteins of *M. gallisepticum* strain S6 (GenBank ID: AFFR01000000).

### 2.4 Data-independent acquisition

Data was obtained by triplicate injection of each sample with LC parameters and configuration identical to IDA experiments, 32 overlapping SWATH windows covered the mass range of 400–1000 Da (single window width – 18.8 Da). Collision energy was the same for all SWATH windows and a ramp was performed from 25 to 40 V for each spectrum. General data analysis is described in [4].
