# Supplementary material for: Data-independent proteome profile of Mycoplasma gallisepticum under normal conditions and heat stress
Source: Data Brief. 2017 Dec 7;16:700–4. doi: 10.1016/j.dib.2017.11.093 (PMC5847720; doi:10.1016/j.dib.2017.11.093)
Supplement: Supplementary file 1 — Transparency document [file mmc1.docx]

Authors declare no conflict of interest.
